# “More bang for the buck”: exploring optimal approaches for guideline implementation through interviews with international developers

**DOI:** 10.1186/1472-6963-12-404

**Published:** 2012-11-15

**Authors:** Anna R Gagliardi

**Affiliations:** 1 University Health Network, 200 Elizabeth Street, Toronto, Ontario, Canada

**Keywords:** Guideline development, Guideline implementation, Qualitative research

## Abstract

**Background:**

Population based studies show that guidelines are underused. Surveys of international guideline developers found that many do not implement their guidelines. The purpose of this research was to interview guideline developers about implementation approaches and resources.

**Methods:**

Semi-structured telephone interviews were conducted with representatives of guideline development agencies identified in the National Guideline Clearinghouse and sampled by country, type of developer, and guideline clinical indication. Participants were asked to comment on the benefits and resource implications of three approaches for guideline implementation that varied by responsibility: developers, intermediaries, or users.

**Results:**

Thirty individuals from seven countries were interviewed, representing government (n = 12) and professional (n = 18) organizations that produced guidelines for a variety of clinical indications. Organizations with an implementation mandate featured widely inconsistent funding and staffing models, variable approaches for choosing promotional strategies, and an array of dissemination activities. When asked to choose a preferred approach, most participants selected the option of including information within guidelines that would help users to implement them. Given variable mandate and resources for implementation, it was considered the most feasible approach, and therefore most likely to have impact due to potentially broad use.

**Conclusions:**

While implementation approaches and strategies need not be standardized across organizations, the findings may be used by health care policy makers and managers, and guideline developers to generate strategic and operational plans that optimize implementation capacity. Further research is needed to examine how to optimize implementation capacity by guideline developers, intermediaries and users.

## Background

Guidelines are syntheses of best available evidence that, in addition to professional judgment and patient preferences, support decision-making by clinicians, managers and policy makers about the organization, delivery and improvement of health care [1,2]. However, population-based studies continue to show that guidelines produced worldwide by prominent agencies for chronic and acute conditions are underused [3-7]. Modeling by the World Health Organization found that for cancer, a third of cases could be prevented, another third cured, and the rest effectively managed if care consistently complied with existing guidelines [8]. The same may be true of many other conditions that feature low compliance with guideline-recommended care, and which impose considerable burden on individuals and health systems.

It is imperative that we improve, or seek new ways of promoting guideline use, but there are many possible ways to do this. To identify the most productive avenue for ongoing investigation it is essential to first analyze related issues, and select an approach that is relevant and actionable. Many factors influence whether and how guidelines are used, including guideline characteristics (quality of format and content), and individual (provider characteristics), institutional (capacity to collect, adapt, share and apply evidence), and health system (policies, resources) attributes [9-12]. While these factors may challenge use of guidelines, it appears that a number of issues may be influential further upstream of the setting in which guidelines are meant to be applied.

Repeat survey of Canadian guideline developers in 1994, 2002 and 2008, and a survey of organizations in Africa, Asia, Europe, Latin America, the United States, and United Kingdom found that few actively implemented their guidelines [13,14]. There may be several reasons for this. Evidence on the impact of different guideline implementation strategies is unclear. Educational (materials, meetings), social engagement (opinion leaders, educational outreach), embedding (clinical support systems, reminders), and incentive (audit & feedback, pay-for-performance) approaches had a small to moderate impact either alone or in combination, but not consistently, and many of these studies did not include economic analyses [15-22]. Most strategies are not routinely applied outside of experimental studies and few program evaluations are published which would provide information about the impact and implications of these strategies in a variety of contexts. By systematic review we found no studies evaluating implementation of breast cancer management guidelines [23], and only two studies evaluating implementation for colorectal cancer management guidelines [24]. Therefore, little data about cost and contextual effectiveness is available to guide decisions about which implementation strategies are most useful in the real world.

Guideline implementation is quite complex, involving multiple stages including creating actionable products, adapting them to local context, assessing barriers, selecting and applying interventions, monitoring use, evaluating outcomes, and sustaining use [25]. Interviews with health professionals in the United Kingdom revealed they had difficulty in acquiring resources to fund implementation, often turning to “soft” money from pharmaceutical companies or charities for more traditional passive (mailing, posting on web site) or educational (meetings) strategies [17]. Thus implementation capacity, which could be defined as the knowledge, resources, infrastructure and practices required to implement guidelines may be lacking, and therefore limits use of, or successful application of existing strategies for promoting guideline uptake.

This research suggests that to improve guideline use, we may need to better implement them, perhaps by enhancing the implementation capacity of guideline developers or their sponsors. In other research guideline developers said they did not actively implement their guidelines, but these interviews did not thoroughly explore why, or their views about approaches and resources that could enhance their implementation capacity [14]. Consultation with guideline developers would explore whether this was at all feasible and, if so, how, thereby more clearly identifying promising avenues for research and practice on implementation capacity, or potentially identifying alternative options. Such information is essential if we are to improve guideline implementation and use. This research was conducted to learn about the implementation capacity and activities of representatives from guideline development agencies, and solicit their recommendations for improving implementation capacity or for alternative approaches by which to promote guideline use. While the views of guideline users are important and could differ from those of guideline developers, interviews with users were beyond the scope of this study, and will be pursued in future research.

## Methods

### Approach

Telephone interviews were conducted with representatives of guideline development agencies to learn more about current and recommended implementation capacity, or other strategies for improving guideline use. Qualitative methods were employed using a pragmatic blended design that included a descriptive approach to describe factual information directly conveyed by participants such as current implementation resources and activities, and a grounded approach to elicit and understand participant views about optimizing implementation capacity or other approaches for improving guideline use [26,27]. Rigour was optimized by sampling from a range of guideline development agencies featuring varying characteristics that could influence their practices or views, exploring responses inductively for emerging ideas based on a conceptual framework, extending the conceptual framework by thoroughly examining emerging themes including deviant cases, demonstrating responses from an array of respondents by including an anonymous identification code with exemplary quotes, and comparison of independent thematic coding across two individuals [28]. The University Health Network Research Ethics Board approved this research, and participants provided informed consent prior to their interview.

### Conceptual framework

Given that guideline implementation is complex, the expertise and activities required to implement guidelines differs from the expertise and activities required to develop them. We interviewed professionals who fund, manage and deliver health services, who said they lacked knowledge about how to implement guidelines [29]. They also expressed confusion about responsibility for guideline implementation. Guideline developers who were interviewed by others said that it was the responsibility of users to implement guidelines [14]. Thus it is currently not clear who does and should bear this responsibility. Options include guideline developers or sponsors who may be government agencies, academic institutions or professional societies; guideline users including clinicians, managers or policy-makers; or an intermediary individual or agency whose role it is to transfer guidelines from developers to users [30]. It is not known whether there may be other entities that could or should be responsible for guideline implementation. Accountability for implementation may differ by jurisdiction and organization depending on the health care system and how guideline programs are funded. Any discussion about guideline implementation and associated capacity must be framed according to locus of responsibility. While different entities might bear this responsibility, the preliminary conceptual framework organized these into three broad categories: developers, intermediaries and users.

### Sampling and recruitment

Guideline development agencies were identified in the National Guideline Clearinghouse, a comprehensive inventory of international guidelines maintained by the United States Agency for Healthcare Research and Quality (http://www.guideline.gov). Purposive sampling was used to recruit participants representing guideline development agencies whose perspective may have varied by country, type of developer (ie. government, professional society), or guideline topic focus (ie. specific clinical area, broad range of guideline topics). Within this sampling frame we also selected agencies who had produced guidelines within the most recent five years, and had produced at least five guidelines overall so as to possess sufficient experience to comment on implementation capacity resources and activities. We also sampled agencies likely to have English-speaking representatives so that it was feasible for us to conduct interviews and analyse interview transcripts given limited resources that precluded the use of interpreters. Detailed information from representative, rather than a large number of participants is needed in qualitative research. Sampling was concurrent with data collection and analysis, and proceeded until no further unique themes emerged (thematic saturation). This was determined by prospective review of transcripts and discussion between the interviewer and principal investigator. The individual most responsible for implementation was identified on each guideline agency web site and contacted by email. Non-responders were contacted again by email after two weeks, then by telephone after another two weeks.

### Data collection

A semi-structured interview guide was developed and pilot tested on two guideline developers to refine wording and flow of questions. Interviews were conducted by a research coordinator with experience in qualitative data collection, who was further trained and mentored by the principal investigator through all phases of the study. Participants were asked to clarify their definition of implementation, and describe how their organization currently implemented guidelines including mandate and resources. They were then asked to comment on the benefits and resource implications of three different approaches to guideline implementation that varied according to onus of responsibility: enhance capacity of guideline developers to also undertake implementation, engage facilitative intermediaries to implement guidelines, or enable users to themselves implement guidelines by including implementation instructions within guidelines. Participants were also asked if apart from these three options they would recommend alternative options or entities with responsibility for implementing guidelines. Telephone interviews of approximately 30 minutes were audio-recorded, and converted to text by a professional transcriptionist.

### Data analysis

Unique themes were identified in an inductive manner using qualitative analysis (31,32). Transcribed narrative was read independently by two individuals to identify, define and organize themes. A log was maintained of emerging codes, their definition, and narrative illustrating application of that code (open coding). The narrative was reviewed several times (constant comparative technique) to identify all instances of the coding framework, and items not matching the framework, to determine whether and how to expand or merge thematic codes (axial coding). The two individuals compared findings and achieved consensus through discussion. Coded text was tabulated by question, theme, and type of producer and country to identify trends. Findings were analysed to identify implications, and confirm and extend the conceptual framework.

## Results

### Participants

Sixty-eight guideline development agencies were contacted, of which 38 either declined to participate or did not respond. Individuals from 30 agencies in seven different countries participated in interviews, 12 from government agencies (including the World Health Organization) and 18 from professional societies (Table 1). These organizations produced guidelines for a variety of disciplines (ie. anaesthesiology, cardiology, neurology, nursing, psychiatry, radiology, urology, etc.) and clinical indications (ie. cancer, hypertension, osteoporosis, stroke, etc.). No trends in responses were identified by country, type of developer organization, or guideline clinical indication. Key themes are discussed below and supported with exemplary quotes from a range of participants.

**Table 1:** Interview participants

**Country**	**Type of organization**		**Total**
	**Government**	**Professional society**	
Australia	3	1	4
Canada	4	11	15
Finland	—	1	1
Netherlands	1	—	1
New Zealand	1	—	1
United Kingdom	2	—	2
United States	—	5	5
WHO	1	—	1
Total	12	18	30

### Implementation is both an activity and an outcome

To establish a common understanding of the purpose of the interview, participants were asked to discuss the meaning of implementation. All participants understood implementation to be both an activity and an outcome of that activity. Implementation referred to a variety of activities employed by guideline developers, from making guidelines accessible to users, to disseminating guidelines to users, and more actively promoting and supporting their use. In this sense, implementation was undertaken: *to reach the right audiences via a range of media and approaches* (06). Implementation was also interpreted as an outcome, which was understood by all participants as: *uptake of guidelines into clinical practice, or into policy that influences practice* (16).

### Implementation capacity varies across developers

To learn about implementation capacity participants were asked to describe their mandate, knowledge, and resources for implementing guidelines. Responses were mixed when participants were asked if their organization was responsible for implementing guidelines. Some organizations had a clear mandate for guideline implementation: *we have a mandate to encourage and support clinicians to use our guidelines* (21). Those that did not have an implementation mandate said that: *the responsibility lies with health care professionals and the health care structure* (15).

Funding for implementation was lacking across most guideline developers. In some organizations no funds were available so guidelines were neither disseminated nor implemented: *There are no funds for dissemination* (26). In other organizations with few resources some guideline sharing activities were achieved with: *whatever funding has been raised…mainly through corporate sponsorship* (06). Even organizations with a mandate to promote use of their guidelines lacked dedicated funding: *it’s built into the budget for doing the guideline* (12).

As a result of limited funding, most organizations had few staff dedicated to implementation. Staffing models for carrying out implementation appeared to vary widely across organizations. In some organizations internal staff managed guideline sharing activities: *we have two people on staff who coordinate the web site and mailing* (10). In other organizations internal staff engaged external health professionals to assist with implementation: *staff work with 25 clinicians on developing and implementing guidelines* (15). In particular, staff dedicated to, and with experience or training for implementation were lacking. Few respondents noted that they employed an implementation specialist: *we have a knowledge translation methodologist* (17).

### Selection of implementation strategies is guideline-dependent

Participants were asked if a framework or model was employed by their organization to make decisions about guideline implementation, or to integrate guideline development and implementation with quality improvement. Most participants noted they did not: *have a standard approach* (09) or *strategic framework* (21), including organizations that had a mandate to undertake implementation. Most participants said that implementation was largely dependent on the nature of the guideline, and that: *not all guidelines receive the same level of implementation effort* (12).

### Most guidelines are disseminated but not implemented

Most guideline developers disseminated their products, which involved making guidelines available to users, often through journal publications or posting them to a web site, or distributing guidelines by regular or electronic mail. A few organizations employed more active implementation strategies including educational workshops, champions or mentors or work groups, academic detailing, and partnerships for advocacy or accountability. The range of implementation activities are summarized in Table 2 with exemplary quotes.

**Table 2:** Implementation activities

**Type of activity**	**Activity**	**Exemplary quotes**
Making guidelines available for users to acquire them	Journals	· Not only in our journal but we’ll try to get it into up to six other professional journals depending on the discipline (06)
		· All of the guidelines are streamlined for peer review publication (12)
		· We produce articles in medical journals (15)
		· We will get it published in a peer reviewed journal as a supplement or summary of the guideline (21)
	Web site	· We post guidelines on our web site (08)
		· The main strategy is our web site (24)
		· We make them available on the Guidelines International Network web site (01)
		· We also submit our guidelines to the Guideline Clearinghouse and the Guidelines International Network (21)
Alerts	Communicated to members	· The bi-annual newsletter includes a page on guidelines (08)
		· We’ve got a newsletter that we circulate quarterly (20)
	Communicated via media	· We do media launches at medical conference (20)
		· Media releases to the medical press (21)
		· Mass media campaign (22)
Distributing guidelines or guideline alerts	Regular mail	· We make about 20,000 copies of our documents and they’re distributed nationally (10)
		· Mailings whenever they’re revised (11)
		· A paper based format is sent out (17)
	Electronic mail	· We send email to our membership (05)
		· We will disseminate an electronic version as widely as possible using extensive email networks (21)
Active promotion and support of guideline use	Educational meetings	· Case-based workshops at our annual meeting (06)
		· We do a lot of workshops (09)
		· Rounds are telecast across the region (13)
		· We host an annual implementation conference where people talk about what they have done and share good ideas and practices (15)
		· Regional and national presentations (26)
	Educational courses	· We set up an online course where clinicians can compare their practice to the guidelines (07)
		· Our web modules include text, videos, and self audit questions (24)
		· We offer short learning courses online that introduce a topic along with multiple choice questions (25)
	Individualized instruction	· Academic detailing (05)
		· Staff meet with care providers to educate them about guidelines (13)
	Champions or mentors	· We have champions in the community (10)
		· We identify champions in each area (15)
		· We have a network of physician champions (17)
	Partnerships for advocacy or accountability	· We worked with hospitals to become an accredited centres of excellence (01)
		· There’s an advocacy side working with government (10)
		· We have expanded programs through the Ministry of Health (17)
		· We work with professional colleges (25)

### Different implementation approaches associated with benefits and limitations

Participants were asked to comment on the implementation capacity implications of three different approaches for improving guideline implementation which differed according to responsibility for implementation: developers, intermediaries, and users.

#### Guideline developers are the only entity with sufficient knowledge of their guidelines

The first option they were asked to consider was enhancing the capacity of guideline developers to also undertake implementation. A few participants thought that it was essential for organizations developing guidelines to also implement them. Some participants expressed this opinion because they believed that no other entity would assume the responsibility: *who else would do it if we didn’t?* (06). Implementation of guidelines by their developers would also maintain continuity: *it’s important for internal consistency to do it in-house all under one roof* (10). This suggests that even if other entities were able to implement guidelines, developers may be better positioned to do so given their knowledge of, and experience with the products, which presumably would facilitate implementation.

#### Guideline developers less effective, more costly than engaging users to implement guidelines

However, more participants thought this was not a useful approach. They said that: *if there’s no ownership it’s a waste of time* (04), and that users: *would have some very good ideas about how to get the product out to the field* (03), suggesting that user involvement may be a more effective means of achieving the outcome of implementation by enhancing awareness and acceptance of guidelines, and that users would identify implementation strategies more likely to be effective. Several participants questioned the feasibility of this approach because: *it would require significant resources* (21). Therefore, while it may seem logical for developers to implement their guidelines, many simply would not have the implementation capacity to do so, and implementation may be more successful if users were engaged in the process.

#### Multiple potential roles for intermediaries in guideline implementation

The second option they were asked to consider was engaging a facilitative intermediary to undertake implementation. Several participants said their organizations were already using this strategy with good results: *we’ve utilized that practice and it’s been very effective* (13). Others not using this approach believed it to be: *a critical strategy* (12) and *a very powerful way to make implementation happen* (15). Thus the overall consensus was that intermediaries were a useful approach. Participants identified a variety of roles for intermediaries, including providing developers with advice on how to implement a guideline: *helps you develop a tailored approach because each setting is very different* (17), taking an active role in implementing a guideline and influencing their peers to adopt the guideline based on their role as leaders: *to implement in the front line you need to have champions within the organization* (01), or by contextualizing or modeling the behaviour embodied in the guideline recommendations: *to see how the guideline can fit in the care pathway based on the knowledge of a peer* (15).

#### Uncertainty about characteristics and cost of intermediaries limit their feasibility

However, diverse opinions were expressed about the characteristics of individuals who would be intermediaries. Some thought the intermediary should be an individual external to intended users of the guideline: *with expertise in knowledge translation* (17) so that they would know how to influence knowledge and behaviour, or a *project manager person* (10), so that they understood change management processes. More participants thought that an intermediary should be: *someone from the professional group whose behaviour you are trying to change* (20), *a physician authority* (01), or *someone with credibility inside the organization* (04), in essence, someone more like, or working closely with the intended users of the guideline who were: *content experts* (12) or: *who have a clinical background* (13). Many viewed this as a feasible approach if intermediaries were individuals who donated their time: y*ou could do it with a network of volunteers* (18). More individuals did not think this was feasible: *it would be ideal if they do it as volunteers but I just don’t see that happening* (26) because volunteers *would have to be paid* (17). Some participants said that, even if intermediaries were volunteers, funding would be required to identify, train and support them: *there needs to be resources to identify these people and to develop those skills* (02). Therefore, while most participants initially said that intermediaries were likely to be an effective approach for implementing guidelines, they were uncertain about how they should be operationalised.

#### Equipping users to implement guidelines in demand, easier and longer-acting

The third option participants were asked to consider was incorporating information within guidelines that would enable users to implement them. Few participants said that their organizations currently include such information in guidelines, but several said that it was a useful approach and they were thinking about it. Most thought it was a good strategy because: *it would be easier to implement [than other strategies] in a shorter amount of time and the effects are lasting because at any point you could access that additional information* (18). Participants also said that: *there is a lot of demand for these additional resources* (15) among users, which would provide them with: *frameworks to guide implementation* (02) and: *bring evidence to them in a very concise way* (16).

#### Enhanced guidelines lengthy, and require skilled staff and additional implementation

Participants not in favour of this strategy thought that regardless of content the guidelines would still need to be implemented: *more information isn’t going to support them in moving it into practice* (07). This suggests that additional strategies may be required to promote use of the guidelines enhanced with user implementation instructions. Participants also thought that additional content would make guidelines lengthy and more difficult to use: *adding more information makes it even less attractive, they haven’t got time to look through it all* (04). From a resource perspective, adding user implementation content to guidelines requires: *staff who have a different skill set to do all of that* (18) which *would be an additional cost* (08). Therefore, while most participants said that this approach was in demand and beneficial, a few participants identified challenges associated with developing and implementing enhanced guidelines.

### No alternative approaches/responsible entities identified

All participants were asked to suggest alternative approaches or responsible entities for implementing guidelines apart from those that were specifically discussed. No additional approaches or entities were identified, thus the conceptual framework was not modified.

### Enhancing guidelines to enable user implementation the preferred approach

Participants were next asked to consider the benefits and challenges of all three guideline implementation approaches and choose the one they thought would be most likely to lead to the implementation and use of guidelines. Most selected the option of including instructions within guidelines so that users could implement them. Several reasons were provided. Adding implementation instructions to guidelines would help users apply the recommendations in practice: *that’s what brings the guideline to life and helps people put the information in practice* (06). Several participants though that relatively speaking, this approach would require the least resources: *that’s the cheapest one because we don’t need so many methodologists* (24). In fact, some stated that this approach would achieve: *more bang for the buck* (26) and, because it was feasible and cost-effective, more likely to have impact because it could be broadly applied: *because it’s more feasible it would be more widespread and therefore more impactful* (26).

### Implications for developing enhanced guidelines that support user implementation

Comparison and analysis of the pros and cons associated with contrasting implementation approaches that differed by onus of responsibility further emphasized that enabling users to implement guidelines by providing them with instructions and tools for doing so represents the most productive avenue for ongoing investigation and practice (Table 3). While insufficient resources were cited as a limitation of all three approaches, empowering users to implement guidelines was considered the least costly. While instructions and tools would need to be created to help guideline developers generate enhanced guidelines, this was thought to be more easily accomplished in less time than expanding the implementation capacity of developers, or identifying, training and supporting intermediaries. Its effect was also viewed as longer lasting because implementation is not a discrete event and instructions and tools would always be available once developed. Furthermore, participants thought that it was more effective to engage users in implementation rather than have an external entity impose guidelines on them, and that users represented the most influential type of intermediary. A few participants said that even enhanced guidelines would need to be implemented. While that may be, what may not have been clear to some participants was that the enhanced guidelines would provide users with tools, templates and instructions to implement them. No other entities were identified that could be responsible for guideline implementation so the conceptual framework was not modified. Given the implications of cost, effort, time, engagement and impact, incorporating implementation instructions and tools in guidelines to enable implementation by users emerged as the most relevant and actionable approach.

**Table 3:** Implications of approaches to guideline implementation that differ by onus of responsibility

**Implications**	**Responsibility**		
	**Developers**	**Intermediaries**	**Users**
Pros	· Continuity with development	· Multiple mechanisms/roles:	· User demand for instructions and tools
	· There is no one else to implement guidelines	○ Advise on tailoring of implementation	· Lasting effect because instructions and tools always available once developed
		○ Influence peers as champions	· Feasible (easier, faster, least costly) so could be widely adopted
		○ Assist with implementation	
Cons	· Insufficient resources are available with which to build capacity	· Clinicians most suitable intermediaries but not likely to volunteer	· Users have limited time to look at more information in guidelines
	· Better to engage users in development and implementation	· Resources needed to identify, compensate, train, and support them	· Resources needed to develop instructions and tools

## Discussion

The importance of guidelines as decision-making tools that promote evidence-informed practice and serve as “one of the foundations for efforts to improve health care” was recently emphasized in a review of the guideline enterprise [31]. Other research has focused on identifying gaps (over/underuse of guidelines) in care, highlighting the need to better implement guidelines, and identifying factors contributing to those gaps [3-11]. Considerable research has also found that many guideline implementation strategies can be effective, but their cost and usefulness in different settings remains unclear [15-23]. Promising research is underway on how to tailor these implementation strategies for various contexts [32]. However, the issue of who bears responsibility for using these strategies to implement guidelines remains, along with the need to better describe and enable the capacity for doing so. Our research fills the unique niche of investigating the feasibility and resource implications of differing implementation approaches according to locus of responsibility and real world circumstances.

We learned that not all guideline development organizations are mandated to implement their guidelines. Those with an implementation mandate featured widely inconsistent funding and staffing models, differing approaches for choosing implementation strategies most often dependent on guideline characteristics, and a range of dissemination and implementation activities including access (web sites, publications), alerts (newsletters, media campaigns), distribution (regular mail, email), education, champions, and organizational partnerships. These findings highlight variable implementation practices and emphasize the lack of insight on how to optimize implementation capacity. While implementation approaches and strategies need not be standardized across organizations, the findings suggest that even if we learn more about how to tailor implementation strategies to enhance their effectiveness, it is not clear who would apply these strategies, and how, given variable mandates, lack of frameworks to guide implementation decision making, and limited resources.

It is well recognized that producing guidelines does not improve the quality of health care, yet the issue of how to achieve implementation of guidelines remains. Participants were asked to comment on the pros and cons associated with three implementation approaches that differed by responsibility, and to recommend an optimal approach. Most participants selected the option of including information within guidelines that would help users to implement them. Given variable mandate and resources for guideline implementation, it was considered the most feasible and cost-effective approach, and therefore most likely to have impact due to potentially broad use. Participants referred to this as “more bang for the buck”. While the other two strategies considered were perceived to have some merit, their feasibility and application were questioned, so it is not surprising that an innovative approach was preferred. Many research studies have found that implementation capacity is a key barrier of guideline implementation [1,7], and that, quite reasonably, as a result of limited mandate and resources many developers do not implement their guidelines [13,14,17]. While intermediaries were commonly viewed as an effective mechanism for promoting use of guidelines, uncertainty about their characteristics and roles as expressed by participants was similar to that described in published research [16,17,30].

We are investigating how to tailor guidelines with information that supports user implementation. A review of the medical literature identified features desired by different users, or associated with guideline use [33]. The Guideline Implementability Framework included 22 elements organized within eight domains: adaptability, usability, relevance, validity, applicability, communicability, resource implications, implementation and evaluation. Subsequent analysis of 20 high quality guidelines on various clinical indications found that most did not contain implementability elements, highlighting numerous opportunities to potentially improve guideline development and use by integrating one or more of these elements [33]. We have identified and described tools that could be included in guidelines to help users address Resource Implications (equipment or technology needed; industrial standards; policies governing their use; type and number of health professionals needed to deliver services; education, training or competencies needed by staff to deliver services; anticipated changes in workflow or processes during or after adoption), Implementation (identifying barriers associated with adoption; selecting and tailoring implementation strategies that address barriers) and Evaluation (tools based on performance measures to assess baseline and post intervention compliance with guidelines). These tools will populate an open directory to which others can contribute. This is a core activity of the Guideline Implementability Research and Application Network (GIRAnet), and may lead to a sustainable effort where guideline developers, implementers and researchers contribute to, and draw from this shared resource on an international basis [34].

Interpretation of study results may be confounded by several issues. The limitation most commonly associated with exploratory or qualitative research is transferability or relevance to other settings. We attempted to mitigate this through purposive sampling of 30 guideline development agencies from seven different countries that produced guidelines for a variety of disciplines and clinical indications. While we achieved thematic saturation, meaning that no further unique ideas emerged with successive interviews, and we found no trends by country, type of guideline developer organization, or guideline clinical indication, we sampled only from industrialized, English-speaking countries, which may have introduced selection bias. The notable exception was the World Health Organization which produces guidelines for non-industrialized countries and therefore provided opinions based on relevance of strategies for implementing guidelines in those settings. Another possible limitation is that participants were prompted to discuss only three differing approaches for guideline implementation. While there are many strategies for implementing guidelines, we chose these three high level approaches specifically because they represented a diversity of responsibility for guideline implementation, but each still encompassed a range of possible implementation strategies. Finally, even if capacity were improved to optimize these approaches, a number of contextual barriers challenge guideline use in the settings where health care is delivered. While this is true, the approach of creating implementable guidelines, thought to be most promising by guideline developers and which we are investigating, is meant to help users overcome those contextual barriers. Tailoring of guideline products in this manner may be complementary to tailoring of strategies to implement those guidelines, but further research is needed to develop these approaches, and more rigorously evaluate their individual and combined impact.

To develop and broadly apply the guideline implementability approach, participants highlighted two key implications that must be considered. Guideline developers will need some direction on how to generate guidelines with user tools, templates or instructions for implementation. We found that no guideline development instructional manuals offered advice on how to generate implementable guidelines [35]. Therefore further research is needed to explore the processes and resources used by developers who have produced guidelines containing user implementation tools, templates and instructions, and share this information about processes and resources with other guideline developers via an instructional manual or training opportunities. Two, while enhanced guidelines are meant to provide users with tools, templates and instructions for implementing the recommendations, these enhanced guidelines may become too complex, and may still require promotion to ensure that potential users are aware of them. A meta-review of factors influencing guideline implementation found that complexity of guidelines was the most frequently cited barrier [7]. In particular, the review found that guidelines which are easy to understand, can be easily tried out, and do not require specific resources are more likely to be implemented. Thus while guidelines containing additional tools, templates or instructions to support user implementation may be lengthy, this content is meant to help users more easily accommodate the recommendations by identifying resource implications and implementation strategies. The review also found that effective strategies have multiple components. Thus, supplementing the range of dissemination strategies already used by international guideline developers with guidelines that equip users with tools, templates and instructions to implement them represents an approach that warrants further investigation.

## Conclusions

Given variable mandate and resources, international guideline developers thought that including information within guidelines that would help users to implement them was the most feasible approach, and more likely to promote guideline use due to potentially broad application. These findings are not likely to change policy across jurisdictions that govern resourcing of guideline developers to undertake guideline implementation, but may emphasize the need to evaluate current and future strategic and operational plans that involve health care quality improvement according to whether and how guideline implementation is considered. Guideline developers may also use these findings to launch internal planning of guideline development and implementation approaches, staffing and methods, and as the basis for consultation with funders and end users about how to optimize current strategies. Ongoing research is needed to examine implementation capacity by guideline developers in jurisdictions other than those involved in this study, the role of different facilitative intermediaries, and whether and how users can be better enabled to implement guidelines.

## Competing interests

The authors declare that they have no competing interests.

## Authors’ contribution

ARG conceived the study and its design, acquired funding, coordinated its conduct, and prepared the manuscript.

## Pre-publication history

The pre-publication history for this paper can be accessed here:

http://www.biomedcentral.com/1472-6963/12/404/prepub
